# Case report of an appendiceal neuroendocrine tumor (carcinoid) combined with a parovarian cyst in an adolescent

**DOI:** 10.3389/fonc.2026.1806034

**Published:** 2026-05-01

**Authors:** Yali Tian, Ying Wu, Fang Hao, Xujian Liu, Meng Kong

**Affiliations:** 1 Department of Pediatric Surgery, Binzhou People’s Hospital, Binzhou, China; 2 Department of Anesthesiology, Children’s Hospital Affiliated to Shandong University, Jinan, China; 3 Department of Anesthesiology, Jinan Children’s Hospital, Jinan, China; 4 Department of Pediatric Surgery, Children’s Hospital Affiliated to Shandong University, Jinan, China; 5 Department of Pediatric Surgery, Jinan Children’s Hospital, Jinan, China

**Keywords:** adolescent, appendiceal neuroendocrine tumor, carcinoid, laparoscopy, parovarian cyst

## Abstract

**Background:**

Appendiceal neuroendocrine tumors (NETs) are rare in adolescents and typically an incidental finding, making preoperative diagnosis difficult. We report an exceptional case of an appendiceal NET coexisting with a parovarian cyst in an adolescent, highlighting the diagnostic challenge and management of this rare combination.

**Case report:**

A 16-year-old female presented with a one-day history of migratory right lower quadrant abdominal pain and fever (38.3 °C). Examination showed right lower quadrant guarding with McBurney’s point tenderness and rebound tenderness. Ultrasound revealed a thickened appendix (max diameter 1.23 cm) and a right adnexal anechoic cyst (3.92 × 2.70 × 2.09 cm). Transumbilical single-port laparoscopy demonstrated a congested, edematous appendix with a firm 1.3-cm diameter area 3–4 cm from its base, and a well-defined 4.0 × 3.0 × 2.0 cm cystic mass within the right broad ligament. Appendectomy and parovarian cyst enucleation were performed. Pathology confirmed a well-differentiated neuroendocrine tumor (G1,WHO classification) in the appendiceal muscularis, with positive immunohistochemistry for CD-56, chromogranin A (Cg-A), cytokeratin low molecular weight (CK-LMW),Syn and Ki-67. The parovarian lesion was a serous cystadenoma. The patient recovered well with no recurrence over 7 years of follow-up.

**Conclusion:**

Appendiceal NETs are rare in adolescents, often presenting as acute appendicitis, and are difficult to diagnose preoperatively. Its combination with a parovarian cyst is even rarer. Transumbilical single-port laparoscopic surgery enabled simultaneous diagnosis and treatment in this case.

## 1 Introduction

Appendiceal neuroendocrine tumors (NETs), formerly known as carcinoids, originate from enterochromaffin cells (Kultschitzsky cells) (1). They grows slowly, rarely metastasizes, and are often discovered incidentally during surgery for appendicitis (2). Parovarian cyst are benign cystic lesions located within the mesosalpinx or broad ligament, originating from embryonic remnants (3). The coexistence of an appendiceal NET with a parovarian cyst is rarely reported. This article describes a 16-year-old female who presented with acute appendicitis and was found intraoperatively to have both an appendiceal NET and a parovarian cyst, both successfully resected via transumbilical single-port laparoscopy.

## 2 Case report

Patient information: A 16-year-old female was admitted with “migratory right lower quadrant abdominal pain accompanied by fever for 1 day.” Her past medical, personal, and family histories were unremarkable.

**Figure 1:**
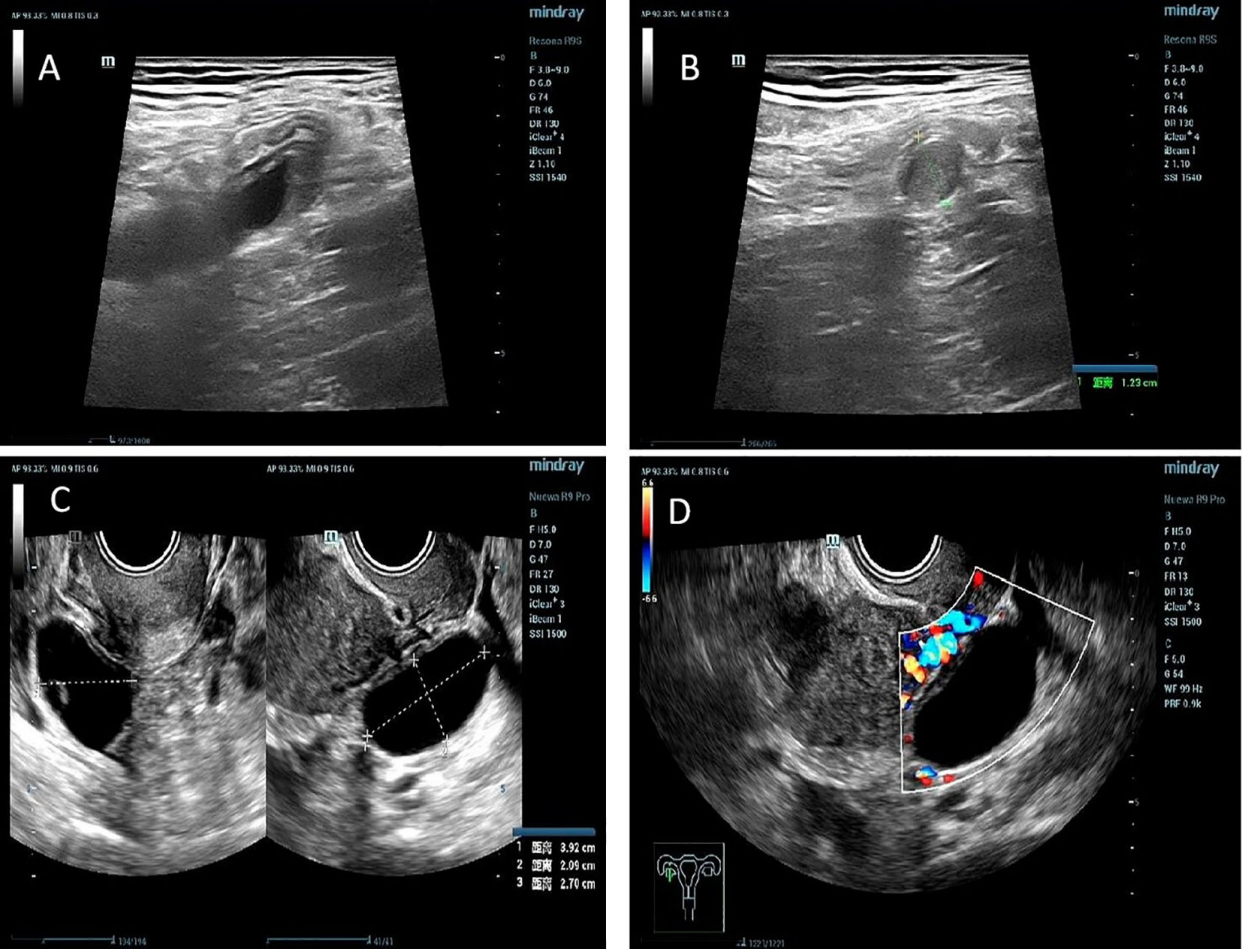
Ultrasound examination of the appendix and parovarian cyst. **(A, B)** Ultrasound revealed significant thickening of the appendix, with a maximum diameter of 1.23 cm and poor anechoic transmission in the lumen. **(C, D)** Anechoic area was detected in the right adnexal region, measuring approximately 3.92 cm × 2.70 cm × 2.09 cm.

Auxiliary examinations: White blood cell count: 8.16×10^9^/L; neutrophil percentage: 69.3%; high-sensitivity C-reactive protein: 17.20 mg/L. Neuron-specific enolase: 18.36 ng/mL; alpha-fetoprotein: 3.55 ng/mL; CA125: 19.19 U/mL. Abdominal ultrasound revealed a thickened appendix (diameter ~1.23 cm) and an anechoic cyst measuring 3.92 × 2.70 × 2.09 cm with clear borders in the right adnexal region (**Figures 1A–D**).

**Figure 2:**
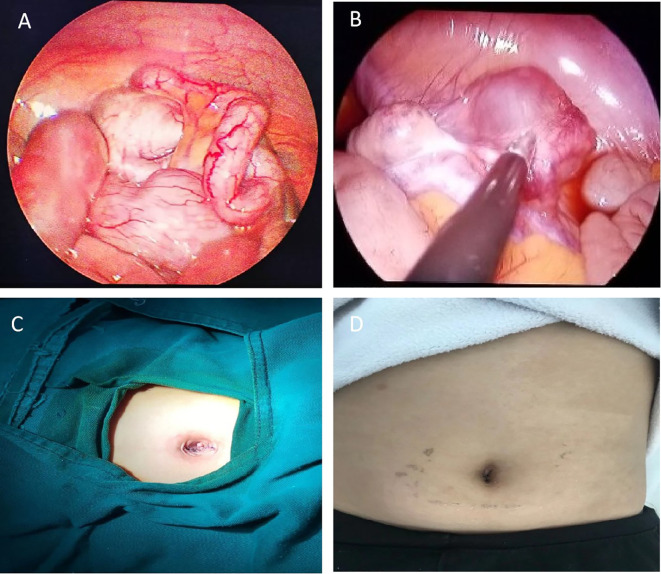
Intraoperative view of the appendix and parovarian cyst and postoperative wound appearance. **(A)** Intraoperative view showing significant thickening of the appendix, measuring 10 cm in length and 1.0 cm in diameter, with congestion, edema, and turbid fluid in the lumen. **(B)** Intraoperative view of the right parovarian cyst, measuring approximately 4.0 cm × 3.0 cm × 2.0 cm, with clear fluid in the lumen. **(C)** Postoperative wound appearance. **(D)** Postoperative wound appearance at 1 month.

**Figure 3:**
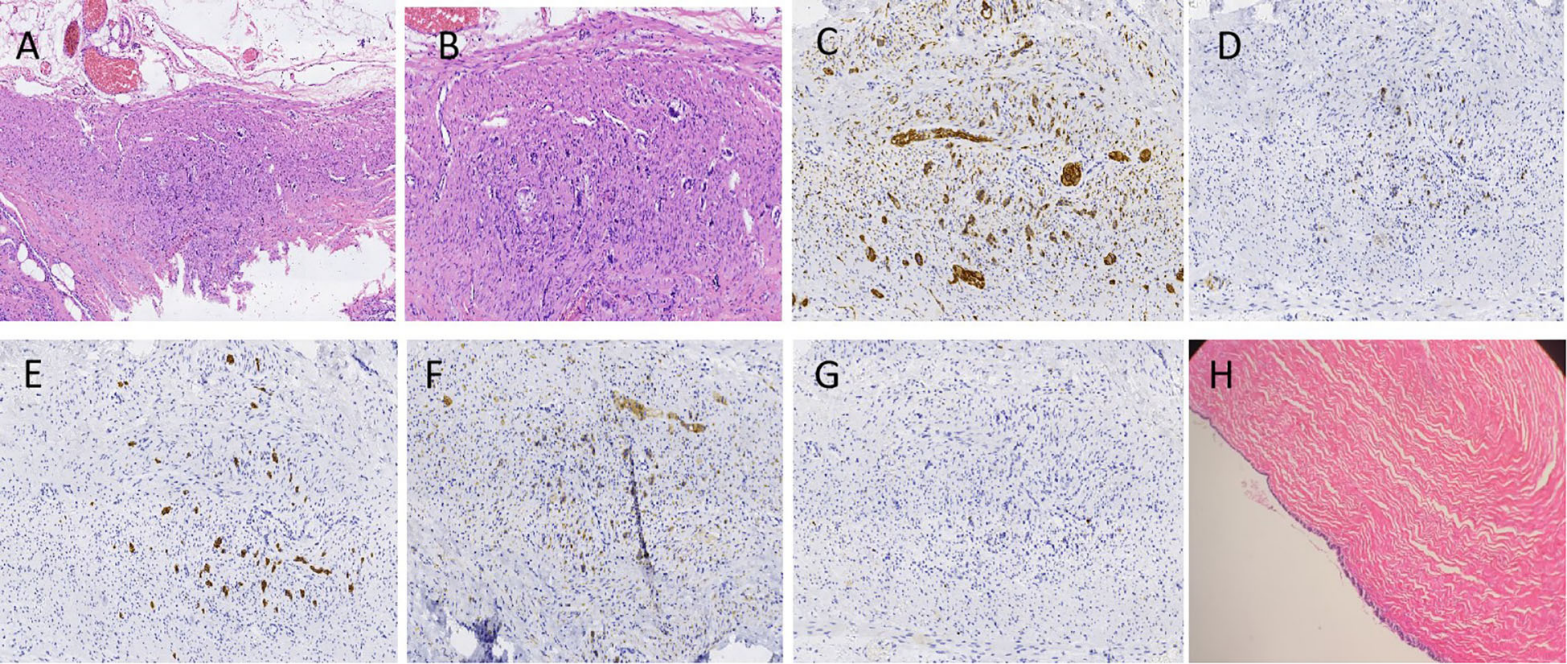
Hematoxylin–eosin (HE) staining and immunohistochemical results. **(A, B)** Microscopic findings: The appendix tumor infiltrates the myenteric nerve plexus and extends into the deep muscular layer but does not reach the serosal surface. The cells are arranged in solid nests or clusters and exhibit cord-like infiltration along the muscular layer. No vascular or lymphatic invasion was observed. The tumor cells are uniform in size and appear round or small and polygonal, with moderate amounts of cytoplasm stained light pink, small round nuclei, and salt-and-pepper chromatin. No obvious nucleoli are observed. The tumor periphery shows retraction, presenting typical carcinoid features. A and B represent magnifications of ×100 and ×200, respectively. **(C–G)** The immunohistochemical results showed that CD-56, Cg-A, CK-LMW, Syn and Ki-67 in the appendix tissue were all positive,×200. **(H)** The parovarian cyst is unilocular, with columnar epithelial cells showing no significant mitotic activity or obvious papillae, ×100.

**Figure 4:**
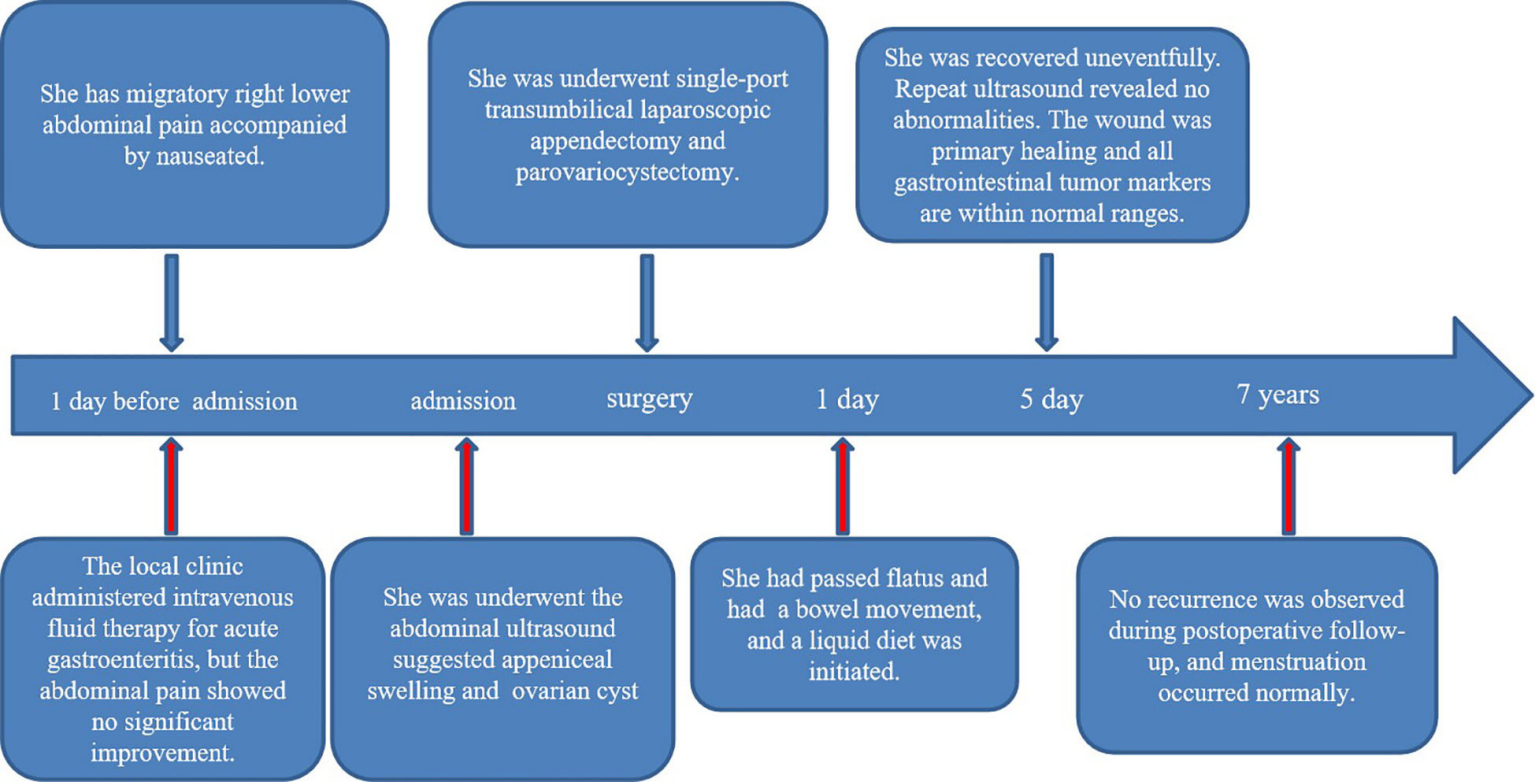
The complete timeline, including diagnosis, surgery, postoperative recovery, and follow-up, was as follows.

Surgery and Pathology: Under general anesthesia, transumbilical single-port laparoscopic surgery was performed. Intraoperatively, the appendix was approximately 10 cm long, congested and edematous, with a firm area approximately 1.0 cm in diameter located 3–4 cm from its base (**Figure 2A**). A cystic mass measuring approximately 4.0 cm × 3.0 cm × 2.0 cm was found within the right broad ligament (**Figure 2B**). After the surgery, the umbilical incision was sutured layer by layer (**Figure 2C**). Appendectomy and parovarian cyst enucleation were performed. The patient was discharged on postoperative day 6.Pathology confirmed an appendiceal NET (diameter approximately 1 mm, G1 grade) (**Figures 3A, B**). Immunohistochemistry was positive for CD-56, Cg-A, CK-LMW, Syn and Ki-67 (Proliferation index < 1%). (**Figures 3C–G**). The parovarian cyst was diagnosed as a serous cystadenoma (**Figure 3H**). The patient recovered smoothly postoperatively, (**Figure 2D**), with no recurrence during 7 years of follow-up (**Figure 4**).

## 3 Discussion

NETs are among the most common neoplasms of the appendix, accounting for approximately 32–57% of all appendiceal tumors (3). They predominantly affect the distal appendix, show a female predominance (female-to-male ratio of 3:1), and are rare in children and adolescents (2). NETs originate from enterochromaffin cells, are neuroendocrine tumors, and can secrete polypeptide hormones (4). Preoperative diagnosis remains challenging, as most patients present with symptoms of acute appendicitis due to tumor obstruction of the appendiceal lumen and are discovered incidentally during surgery (5). This case describes an adolescent female with a distal appendiceal carcinoid tumor and an incidental parovarian cyst. To our knowledge, no case of this specific combination has been reported in the past decade. In our institution, for all cases of suspected appendicitis, we routinely perform ultrasound of both the appendix and the uterine adnexa. Abdominal CT is reserved for patients in whom the ultrasound diagnosis of appendicitis is unclear and further evaluation is needed to rule out other intra-abdominal pathologies. In this case, the parents were concerned about radiation exposure from CT. Because ultrasound clearly identified both the inflamed appendix and the adnexal cyst, no CT was performed. This approach was sufficient for diagnosis and surgical planning.

In an adolescent presenting with right lower quadrant pain, an adnexal mass, and suspected appendicitis, the differential diagnosis includes not only appendiceal pathology but also gynecologic conditions such as ovarian torsion, tubo-ovarian abscess (TOA), and mature cystic teratoma (6, 7). In this case, these entities were systematically excluded based on imaging findings. First, ovarian torsion typically presents with acute pain and an enlarged, edematous ovary with compromised Doppler flow on ultrasound. In this patient, the ipsilateral ovary was visualized separately from the cyst and appeared entirely normal, effectively ruling out torsion. Second, tubo-ovarian abscess usually presents with fever, elevated inflammatory markers, and a complex, thick-walled, septated cystic mass with internal debris on ultrasound. Although our patient had fever and elevated C-reactive protein, the cyst was unilocular, anechoic, and thin-walled, with no surrounding inflammatory changes or free fluid suggestive of abscess formation. Third, mature cystic teratomas often exhibit characteristic sonographic features such as hyperechoic mural nodules, shadowing echogenic foci, or fat–fluid levels. The cyst in this case lacked these features. Its simple, anechoic appearance was most consistent with a benign parovarian cyst. Intraoperative visualization of a normal ipsilateral ovary and a separate broad ligament cyst confirmed this diagnosis.

The extent of surgery for appendiceal NETs should be determined based on tumor size, location, depth of invasion, lymph node status, and patient age. According to the European Neuroendocrine Tumor Society guidelines, simple appendectomy is sufficient for tumors <1 cm in diameter without adverse features such as mesenteric invasion, positive margins, or high mitotic rate (8). For tumors >2 cm in diameter, or those with mesenteric or lymph node metastasis, involvement of the appendiceal base, peritoneal metastasis, or carcinoid syndrome, right hemicolectomy with lymph node dissection is recommended (9). Management of tumors measuring 1–2 cm remains controversial; most experts support right hemicolectomy for tumors located at the base, those with mesenteric or cecal invasion, lymph node metastasis, or pathological diagnosis of goblet cell carcinoid (10). In pediatric patients, a more conservative approach is generally advocated. In this case, the tumor was small (<1 cm) and confined to the muscularis propria, with no evidence of mesenteric invasion, lymph node involvement, or positive margins. Although muscularis propria invasion was noted, current guidelines do not mandate right hemicolectomy based on this finding alone in the absence of other high-risk features (8). Therefore, simple appendectomy with clear margins was considered adequate. The patient did not undergo a secondary right hemicolectomy, and no recurrence has been observed over 7 years of follow-up. This outcome supports the conservative approach in similar cases.

Parovarian cysts, also known as paraovarian or paratubal cysts, are cystic lesions located within the mesosalpinx or broad ligament. They account for approximately 10–20% of adnexal masses and originate from embryonic remnants, including the mesonephric (Wolffian) duct, paramesonephric (Müllerian) duct, or from metaplasia of the tubal serosal epithelium. These cysts are almost always benign serous cysts, with borderline or malignant cases being rare (11). The decision to proceed with surgical enucleation rather than observation in this adolescent patient was based on several factors: (1) the presence of acute abdominal symptoms necessitating surgical exploration for suspected appendicitis; (2) the inability to definitively exclude other adnexal pathologies based on imaging alone; (3) the risk of cyst-related complications such as torsion, hemorrhage, or rupture, particularly during appendiceal inflammation; and (4) the feasibility of concurrent minimally invasive resection via the same transumbilical approach, avoiding a second procedure. Enucleation of the cyst with preservation of the fallopian tube and ovary was performed, which carries an excellent prognosis for future fertility.

Transumbilical single-port laparoscopic surgery successfully resected both the appendiceal carcinoid and the parovarian cyst in a single session. This approach achieved complete excision with minimal invasiveness and a good cosmetic outcome. The patient recovered well with no recurrence over 7 years of follow-up. However, it must be acknowledged that the advantages of this specific technique cannot be generalized from a single case. As a single case report, these findings cannot be generalized. Nevertheless, several practical points emerge for clinical practice: (1) Preoperative ultrasound should systematically evaluate both the appendix and adnexa in adolescent females with suspected appendicitis. (2) Intraoperative frozen section pathology is recommended for appendices with abnormal morphology or texture. (3) For appendiceal NETs <1 cm without adverse features (mesenteric invasion, positive margins, high mitotic rate), simple appendectomy is sufficient; right hemicolectomy is not required. (4) Long-term follow-up is recommended, although the prognosis for small appendiceal NETs is excellent with 5-year survival rates. In this case, the patient has remained disease-free for 7 years, supporting the management decisions made.

## 4 Conclusion

This case highlights a rare combination of an appendiceal NET and a parovarian cyst in an adolescent. The diagnosis was made intraoperatively, as such tumors are difficult to detect preoperatively. Transumbilical single-port laparoscopy enabled simultaneous resection of both lesions with minimal invasiveness. For small, localized appendiceal NETs (G1, <1 cm), simple appendectomy is sufficient, and long-term prognosis is excellent.

## Data Availability

The original contributions presented in the study are included in the article/supplementary material. Further inquiries can be directed to the corresponding authors.
